# Diagnosis and etiologic classification of optic tract lesions

**DOI:** 10.1093/braincomms/fcaf354

**Published:** 2025-09-19

**Authors:** Natalie S Chen, Heather M McDonald, Jonathan Micieli, Edward Margolin

**Affiliations:** Temerty Faculty of Medicine, University of Toronto, Toronto, Ontario M5S 3K3, Canada; Department of Ophthalmology and Visual Sciences, University of Toronto, Toronto, Ontario M5T 3A9, Canada; Department of Ophthalmology and Visual Sciences, University of Toronto, Toronto, Ontario M5T 3A9, Canada; Department of Medicine, Division of Neurology, University of Toronto, Toronto, Ontario M5S 3H2, Canada; Department of Ophthalmology and Visual Sciences, University of Toronto, Toronto, Ontario M5T 3A9, Canada; Department of Medicine, Division of Neurology, University of Toronto, Toronto, Ontario M5S 3H2, Canada

**Keywords:** optic tract lesions, optic tract syndrome, homonymous hemianopia, ganglion cell analysis, optical coherence tomography

## Abstract

Existing literature regarding optic tract syndrome, which arises from lesions affecting the optic tract(s), is limited. The objective of this retrospective cohort study was to elucidate the diverse clinical and imaging manifestations of the optic tract syndrome relative to the causative lesion. The study population comprised of all patients with optic tract syndrome who were seen at two tertiary neuro-ophthalmology practices from 2014 to 2024. Inclusion criteria were (i) signs associated with optic tract syndrome (homonymous hemianopia and/or relative afferent pupillary defect and/or characteristic findings on peripapillary optical coherence tomography or ganglion cell analysis) and (ii) radiographically confirmed optic tract lesion. Patient records were ascertained through medical databases and reviewed. Statistical analysis was performed to identify relationships between clinical/imaging manifestations and lesion type. Fifty-six patients with optic tract syndrome were identified. The mean age was 49.4 ± 16.7 years, and 37 were female patients. Aetiologies included space-occupying lesions (*n* = 25), demyelination (*n* = 11), ischaemia/haemorrhage (*n* = 9), non-specific optic tract atrophy (*n* = 8), perinatal insult (*n* = 2) and trauma (*n* = 1). At presentation, visual field defects were observed in 98% of patients (*n* = 55). Of these patients, 20% (*n* = 11) demonstrated complete field loss while 80% (*n* = 44) demonstrated partial field loss, of which 95% (*n* = 42) were incongruent and 5% (*n* = 2) were congruent. Of the 40 patients with homonymous hemianopia, 78% (*n*  *=* 31) demonstrated incongruous defects. Of the 54 patients with peripapillary optical coherence tomography findings, 41% (*n* = 22) had contralateral band atrophy and ipsilateral hourglass atrophy. Of the 51 patients with available data, 29% (*n* = 15) had contralateral relative afferent pupillary defect. Patients with demyelinating lesions were most likely to present within 2 weeks of symptom onset (*P* = 0.0180) and least likely to exhibit band/hourglass atrophy (*P* = 0.0112). In contrast to previous studies that found a significant percentage of optic tract lesions to produce congruent homonymous hemianopia, most patients in this study demonstrated incongruent homonymous hemianopia. As such, optic tract lesions should be considered in patients with incomplete, incongruous and subtle homonymous defects. Peripapillary optical coherence tomography should be examined for the presence of band and hourglass atrophy in these patients but may be less commonly observed, especially in those with a demyelinating aetiology.

## Teaser

Chen *et al*. examined the diverse clinical and imaging manifestations of optic tract lesions. They found that such lesions should be considered in patients with incomplete, incongruous and subtle homonymous hemianopia. Peripapillary optic coherence tomography should also be used to examine for band and hourglass atrophy.

## Graphical Abstract

**Graphical Abstract:**
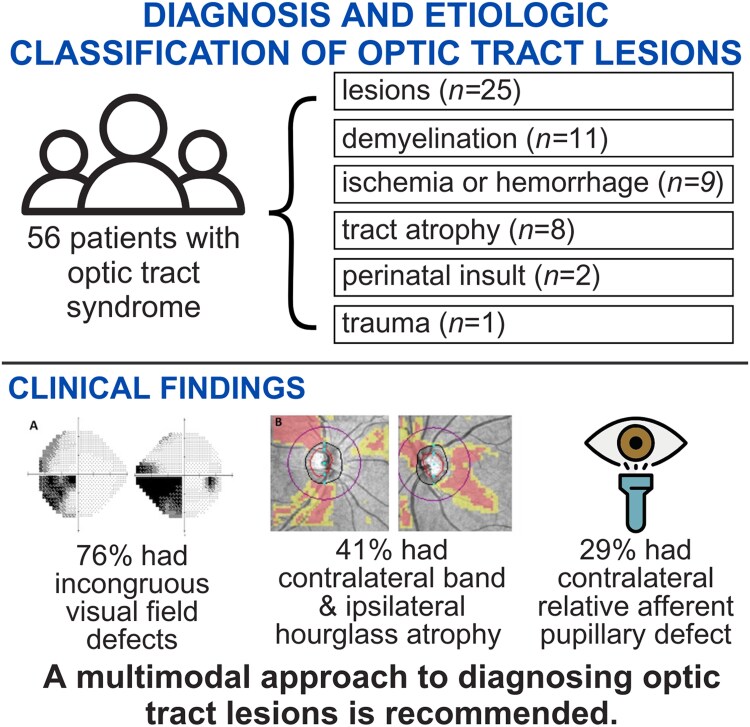

## Introduction

Optic tract lesions (OTLs) are a rare but important cause of visual impairment.^1^ The optic tracts travel from the optic chiasm to the lateral geniculate bodies and can be affected by lesions anywhere along their path.^2^ Possible aetiologies of OTLs include tumours, infarction and demyelinating disease.^1,3^ Optic tract syndrome (OTS) is defined as a combination of contralateral incongruous homonymous hemianopia (HH), presence of mild contralateral relative afferent pupillary defect (RAPD) and contralateral band atrophy along with ipsilateral hourglass atrophy of the optic discs.^4^ More extensive lesions can also present with ipsilateral hemianesthesia and pyramidal tract signs, depending on their location.^4^

Studies reporting presenting features, aetiology and imaging findings of patients with OTLs are limited. There is also a paucity of information characterizing relevant optical coherence tomography (OCT) and ganglion cell analysis findings in this condition. OCT can more clearly demonstrate band and hourglass atrophy of the peripapillary retinal nerve fibre layer (pRNFL) that can be difficult to appreciate clinically. Band atrophy refers to the nasal and temporal pallor of the optic disc, whereas hourglass atrophy indicates refers to superior and inferior pallor. Furthermore, ganglion cell analysis can demonstrate homonymous thinning of the ganglion cell layer (GCL) contralateral to the homonymous field loss seen on perimetry. These findings can be subtle and easily overlooked, leading to missed diagnosis or mismanagement of patients with OTLs. The purpose of this study is to provide a detailed description of neuro-ophthalmic findings, OCT and ganglion cell analysis clues and radiological characteristics of OTLs.

## Methods

This is a retrospective observational study of all patients who were diagnosed with OTLs in two tertiary neuro-ophthalmology practices from January 2014 to January 2024. Study approval was obtained from University of Toronto’s Health Sciences Ethics Board (Protocol #00045646), and all research adhered to the tenets of the Declaration of Helsinki. Electronic charts of all patients with a diagnosis of ‘optic tract lesion’ and ‘homonymous hemianopia’ were reviewed. Patients were included if they were diagnosed with OTS based on the following criteria: (i) presented with signs associated with OTS including incongruous HH and/or RAPD and/or characteristic findings on OCT or ganglion cell analysis and (ii) had a radiographically confirmed OTL. Patients were excluded if they were missing essential clinical or imaging data or had ocular history that obscured clinical presentation.

Data collected for each patient included demographic information (presenting age, sex at birth), time from symptom onset to presentation and presenting symptomatology (vision changes, headache/pain, diplopia or other focal neurological deficits) when available. Clinical data included presenting best-corrected visual acuity, presence of RAPD, extraocular dysmotility, misalignment, papilledema and band/hourglass atrophy of the optic disc(s). Diagnostic imaging information included average pRNFL thickness in each eye, presence of band/hourglass atrophy of the pRNFL and presence of homonymous thinning on ganglion cell analysis. Visual field (VF) data included the pattern of field loss (homonymous quadrantanopia or hemianopia, bitemporal hemianopia or junctional scotoma) and mean deviation in each eye. Patients with bitemporal hemianopia and junctional scotomas were included only when the lesion clearly affected the optic tract but was often large enough to affect the chiasm and/or optic nerve(s) as well. Regarding neuro-radiological findings, the type of imaging used, laterality and location of the lesion and final diagnosis/aetiology of the OTL were recorded. Clinical presentation findings are summarized in Supplementary Table 1.

### Statistical analysis

Most data were presented descriptively with continuous variables being expressed as a mean and standard deviation. Categorical variables were expressed as numbers (*n*) and proportions (%). For further analysis, patients were grouped by their OTL aetiology to identify any differences in presenting characteristics, exam findings or imaging manifestations to the remaining participants. Statistical analysis was performed using MATLAB, with sample code provided in the Supplementary Materials. Fisher’s exact test was used to identify statistically significant differences for categorical variables between groups and the Kruskal–Wallis test was employed to compare differences in the distribution of continuous variables between groups. The Bonferroni correction was applied to minimize Type I error owing to multiple comparisons. For Fisher’s exact test, *P*-values were multiplied by the number of comparisons to obtain the Bonferroni-adjusted *P*-values, with significance set at *P* < 0.05. For the Kruskal–Wallis test, the adjusted significance threshold was calculated by dividing 0.05 by the total number of comparisons, and the *P*-values were compared to this threshold.

## Results

Fifty-six patients were identified, with clinical presentation findings summarized in Supplementary Table 1. Sixty-six percent of patients were female (*n* = 37), with age ranging from 19 to 83 years (mean 49.4 ± 16.7). Patients with space-occupying lesions and optic tract atrophy were, on average, older than 50 years, whereas those with other aetiologies tended to be younger. The average time from symptom onset to presentation was 46.6 ± 177.5 weeks. Patients with demyelinating disease had the shortest average time to presentation (9.3 ± 15.3 weeks), whereas those with non-specific atrophy had the longest delay (313.3 ± 554.3 weeks). Patients presented with various symptoms including bilateral vision loss (*n* = 54; 96%), unilateral vision loss (*n* = 2; 4%), diplopia (*n*  *=* 4; 7%), headache/pain (*n* = 10; 18%) and focal neurological symptoms (*n* = 18; 32%). Headaches were most frequently reported in patients with perinatal insult (50%; 1 of 2 cases) and space-occupying lesions (24%; 6 of 25 cases). Of the 18 patients with focal neurological symptoms, 28% (*n* = 5) had hemiparesis, 28% (*n* = 5) had ptosis, 28% (*n* = 5) had paraesthesia in the face or extremities, 11% (*n* = 2) had dysdiadochokinesia, 11% (*n* = 2) had other symptoms of cerebellar dysfunction, 11% (*n* = 2) had a cranial nerve defect, 11% (*n* = 2) had muscle weakness, 6% (*n* = 1) had nystagmus, 6% (*n* = 1) had hyperreflexia and increased tone, 6% (*n* = 1) had upward gaze palsy, 6% (*n* = 1) had disorientation, 6% (*n* = 1) had hemispacial neglect and 6% (*n* = 1) had alexia without agraphia. Furthermore, most of these patients (94%; *n*  *=* 17) demonstrated additional lesions on imaging that likely accounted for these neurological findings. Focal neurological deficits were most prevalent in patients with an ischaemic or haemorrhagic aetiology (56%; 5 of 9 cases). Nine percent (*n* = 5) were identified incidentally, either through MRI findings or referral for an unrelated ophthalmological condition. Relationships between lesion aetiology and presenting characteristics were examined (Table 1), with the only statistically significant finding being that patients with demyelinating disease were most likely to present within 2 weeks of symptom onset (*P* = 0.0180).

**Table 1:** Characteristics based on type of lesions

Type of lesion affecting the optic tract	Age (years)		Female gender			Time from symptom onset to presentation (weeks)		Patients presenting within 2 weeks of symptom onset			Patients presenting with headache			Patients with focal neurological deficit			Lesion found incidentally		
	Mean ± SD	*P-*value^a^	*n*	%	*P*-value^b^	Mean ± SD	*P*-value^c^	*n*	%	*P*-value^b^	*n*	%	*P*-value^b^	*n*	%	*P*-value^b^	*n*	%	*P*-value^b^
Space-occupying lesion (*n* *=* 25)	54.4 ± 18.0	0.0590	19/25	76	1.000	19.1 ± 25.8	0.803	4/18	16	1.000	6/25	24	1.000	6/25	24	1.000	2/25	8	1.000
Demyelinating disease (*n* *=* 11)	44.2 ±13.6	0.261	7/11	64	1.0000	9.3 ± 15.3	0.0130	6/9	55	**0**.**0180**	1/11	9	1.000	3/11	27	1.000	0/11	0	1.000
Ischaemic/haemorrhagic OTS (*n* *=* 9)	44.7 ± 17.6	0.390	8/9	89	0.8753	30.1 ± 36.3	0.162	0/7	0	0.625	1/9	11	1.000	5/9	56	0.773	1/9	11	1.000
Atrophic OTS (*n* *=* 8)	52.0 ± 12.9	0.574	2/8	25	0.0845	313.3 ± 554.3	0.0377	0/4	0	1.000	1/8	13	1.000	3/8	38	1.000	1/8	13	1.000
Perinatal insult (*n* *=* 2)	33.5 ± 10.6	0.145	1/2	50	1.0000	N/A^d^	N/A	N/A^d^	N/A	N/A	1/2	50	1.000	1/2	50	1.000	2/2	100	0.0818
Traumatic OTS (*n* *=* 1)	37.0 ± 0	0.439	0/1	0	1.0000	N/A^d^	N/A	N/A^d^	N/A	N/A	0/1	0	1.000	0/1	0	1.000	1/1	100	0.750

*P*-values represent comparisons between each lesion aetiology and the remaining patients (i.e. one-versus-all comparison). These were conducted using Fisher’s exact test for categorical variables and Kruskal–Wallis test for continuous variables. Bonferroni correction was applied for all analyses, with significance levels specified below. Bolded values indicate significance.

^a^Significance level after Bonferroni correction: *P* < 0.00833.

^b^Significance level: *P* < 0.05 (*P-*values adjusted for Bonferroni correction).

^c^Significance level after Bonferroni correction: *P* < 0.0125.

^d^All patients were identified incidentally.

### Examination and diagnostic imaging findings

Patients had an average best-corrected visual acuity of logMAR 0.20 ± 0.33 in the eye ipsilateral to the lesion and logMAR 0.17 ± 0.29 in the contralateral eye. An RAPD was detected on the contralateral side of the OTL in 29% (*n* = 15) of 51 patients with available data. Of these patients, 80% (*n* = 12) demonstrated hemianopia, 13% (*n* = 2) quadrantanopia and 7% (*n* = 1) had a junctional defect. The relationship between lesion aetiology and presence of RAPD was also investigated (Table 2), with no association being found. Eighteen percent (*n* = 7) of the 39 patients with extraocular motility data demonstrated dysmotility, while 32% (*n* = 11) of the 34 patients with alignment data had misalignment. Finally, papilledema was observed in 4% (*n* = 2) of all patients, with one case secondary to stroke and the other to trauma. The remainder of patients demonstrated either normal optic discs or pallor, as described below.

**Table 2:** Presence of physical examination and formal perimetry findings based on type of lesion

Type of lesion affecting the optic tract	Presence of RAPD			VF defect											
				Incomplete incongruous homonymous			Incomplete congruous homonymous			Complete homonymous			Other field defect (bitemporal or junctional)		
	*n*	%	*P*-value^a^	*n*	%	*P*-value^a^	*n*	%	*P*-value^a^	*n*	%	*P*-value^a^	*n*	%	*P*-value^a^
Space-occupying lesion (*n* *=* 25)	13/23	57	1.000	17/25	68	1.000	0/25	0	1.000	6/25	24	1.000	2/25	8	1.000
Demyelinating disease (*n* *=* 11)	3/10	30	1.000	7/10	70	1.000	1/10	10	1.000	1/10	10	1.000	1/10	10	1.000
Ischaemic/haemorrhagic OTS (*n* *=* 9)	5/8	63	1.000	8/9	89	1.000	0/9	0	1.000	1/9	11	1.000	0/9	0	1.000
Atrophic OTS (*n* *=* 8)	1/7	14	0.621	5/8	63	1.000	0/8	0	1.000	2/8	25	1.000	1/8	13	1.000
Perinatal insult (*n* *=* 2)	1/2	50	1.000	1/2	50	1.000	1/2	50	0.432	0/2	0	1.000	0/2	0	1.000
Traumatic OTS (*n* *=* 1)	1/1	100	1.000	1/1	100	1.000	0/1	0	1.000	0/1	0	1.000	0/1	0	1.000

*P*-values represent comparisons between each lesion aetiology and the remaining patients (i.e. one-versus-all comparison). These were conducted using Fisher’s exact test. Bonferroni correction was applied for all analyses, with significance levels specified below.

^a^Significance level: *P* < 0.05 (*P-*values adjusted for Bonferroni correction).

### Formal perimetry findings

VF defects were identified in 98% (*n* = 55) of patients at presentation, with most (73%, *n* = 40) demonstrating HH. Of the 40 patients, 95% (*n* = 38) had unilateral HH and 5% (*n* = 2) had bilateral HH. Seventy-eight percent (*n*  *=* 31) of patients with HH demonstrated incongruent defects. Homonymous quadrantanopia was seen in 20% (*n* = 11); of these patients, 64% (*n* = 7) had inferior quadrantanopia while 36% (*n* = 4) had superior quadrantanopia. One patient (2%) exhibited a superior bitemporal field defect due to progressive atrophy of bilateral optic tracts and optic chiasm secondary to presumed hereditary process. Three patients (6%) demonstrated junctional scotoma due to a demyelinating lesion, haemorrhagic lesion or meningioma affecting the optic tract, chiasm and optic nerve simultaneously. The only patient with normal VFs had a demyelinating lesion. Of all patients with VF defects, 20% (*n* = 11) demonstrated complete field loss and 80% (*n*  *=* 44) demonstrated partial field loss, of which 95% (*n* = 42) were incongruent and 5% (*n*  *=* 2) were congruent. No statistically significant associations were determined between lesion aetiology and type of VF defect (Table 2). Average mean deviation was −11.29 ± 8.05 and −9.27 ± 6.64 for the ipsilateral and contralateral eye, respectively.

### OCT and ganglion cell analysis findings

Of the 54 patients with reliable peripapillary OCT findings, 41% (*n* = 22) exhibited the typical signs of OTS characterized by contralateral band and ipsilateral hourglass atrophy. Additionally, 7% (*n*  *=* 4) demonstrated band atrophy only, 6% (*n*  *=* 3) had normal pRNFL and 4% (*n* = 2) had hourglass atrophy only. The remainder (*n*  *=* 23) had non-specific findings. The average pRNFL thickness was 76.5 ± 17.6 µm in the ipsilateral eye and 78.1 ± 13.9 µm in the contralateral eye. Statistical analysis revealed that patients with demyelinating disease were the least likely to exhibit band or hourglass atrophy on pRNFL compared to other groups (*P* = 0.0112). This finding remained significant when limiting the analysis to the 29 individuals who presented within 6 months of symptom onset (*P*  *=* 0.0379) (Table 3). A sensitivity analysis comparing pRNFL findings in patients who presented within and after 6 months of symptom onset was also conducted. Among the patients who presented ≤6 months from onset, 34% (*n*  *=* 10) had pRNFL findings typical of OTS compared to 58% (*n* = 7) of 12 patients who presented >6 months. This difference was not statistically significant (*P* = 0.184).

**Table 3:** Presence of OCT and ganglion cell analysis findings based on type of lesion

Type of lesion affecting the optic tract	Presence of band or hourglass atrophy on OCT RNFL			Presence of band or hourglass atrophy in patients presenting ≤6 months of symptom onset			Presence of homonymous thinning on ganglion cell analysis corresponding to VF defect		
	*n*	%	*P*-value^a^	*n*	%	*P*-value^a^	*n*	%	*P*-value^a^
Space-occupying lesion (*n* *=* 25)	14/24	58	1.000	8/13	62	1.000	6/21	29	0.126
Demyelinating disease (*n* *=* 11)	1/11	9	**0**.**0112**	0/7	0	**0**.**0379**	5/10	50	1.000
Ischaemic/haemorrhagic OTS (*n* *=* 9)	5/9	56	1.000	2/5	40	1.000	5/7	71	1.000
Atrophic OTS (*n* *=* 8)	5/7	71	1.000	1/1	100	1.000	4/5	80	1.000
Perinatal insult (*n* *=* 2)	2/2	100	1.000	2/2	100	1.000	1/2	50	1.000
Traumatic OTS (*n* *=* 1)	1/1	100	1.000	1/1	100	1.000	1/1	100	1.000

*P*-values represent comparisons between each lesion aetiology and the remaining patients (i.e. one-versus-all comparison). These were conducted using Fisher’s exact test. Bonferroni correction was applied for all analyses, with significance levels specified below. Bolded values indicate significance.

^a^Significance level *P* < 0.05 (*P-*values adjusted for Bonferroni correction).

Of the 46 patients with available ganglion cell analysis results, 48% (*n* = 22) demonstrated homonymous thinning, 32% (*n* = 15) had non-specific thinning, 9% (*n* = 4) had junctional thinning (characterized by central/diffuse thinning in one eye and temporal thinning in the other), 9% (*n* = 4) had normal findings, and 2% (*n* = 1) had monocular diffuse thinning. There was no observed association between lesion aetiology and the proportion of ganglion cell analysis corresponding with VF defect (Table 3), although patients with ischaemic or haemorrhagic lesions had the highest proportion overall.

Overall, 16% (*n* = 9) of patients demonstrated both band and hourglass pRNFL thinning and homonymous thinning of GCL on OCT. Forty-six percent (*n* = 26) exhibited only one of these findings, with 23% (*n*  *=* 13) demonstrating typical band and hourglass pRNFL thinning and 23% (*n* = 13) demonstrating homonymous thinning. Thirty-eight percent (*n* = 21) had no corresponding findings. Of the 13 patients that did not have incongruous VF defects, 15% (*n*  *=* 2) demonstrated both pRNFL and GCL thinning, 31% (*n* = 4) exhibited only pRNFL thinning and 23% (*n* = 3) demonstrated only GCL thinning. The remainder (*n* = 4) did not have any corresponding findings.

**Figure 1:**
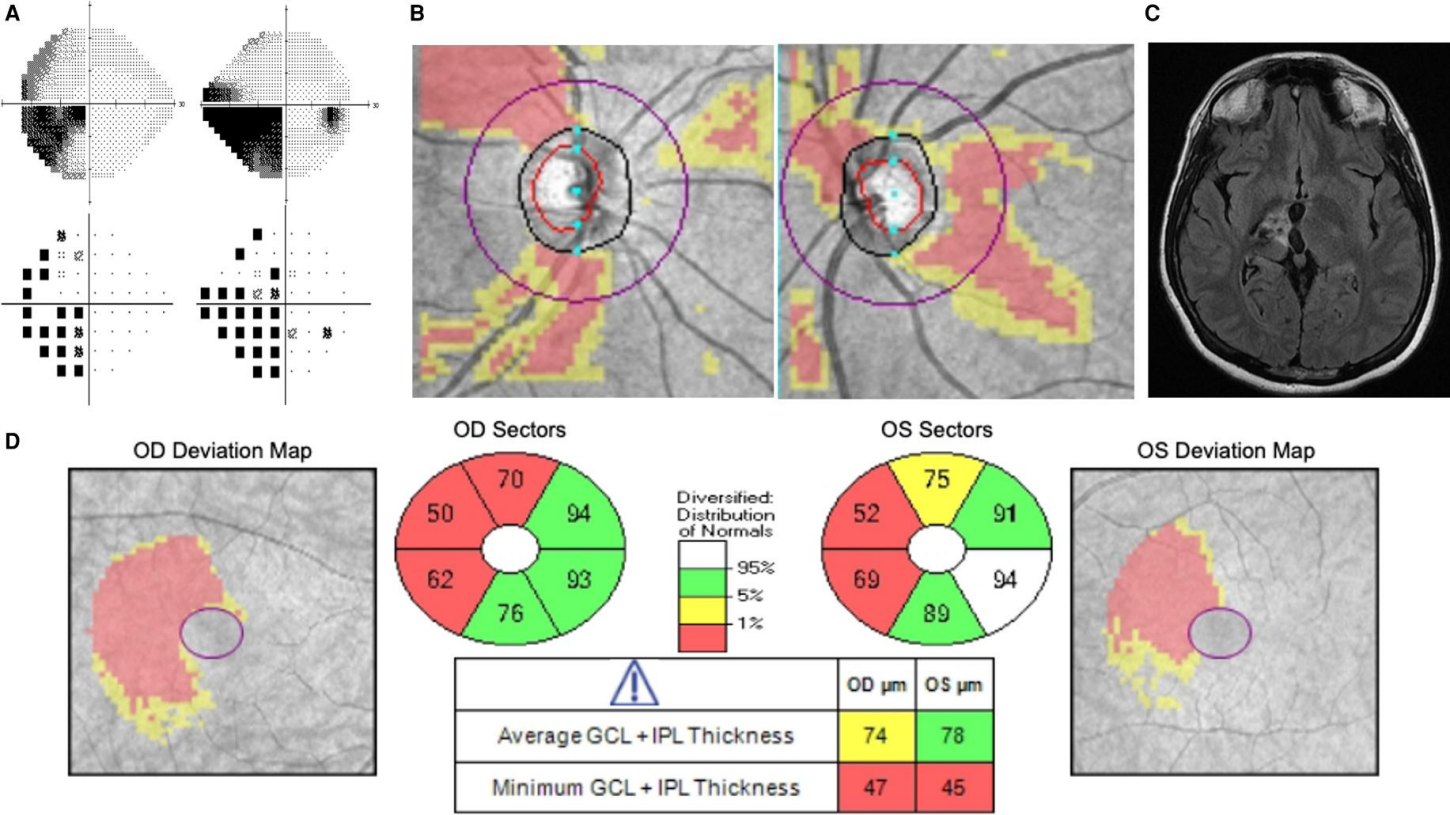
**Optic tract syndrome.** (**A**) Incomplete incongruous left HH seen on greyscale map and pattern deviation map, (**B**) hourglass atrophy of the right optic disc and band atrophy of the left optic disc, (**C**) post-radiation cystic changes following treatment of right thalamic arteriovenous malformation affecting the right optic tract (MRI axial FLAIR) and (**D**) right homonymous thinning of the GCL and inner plexiform layer (IPL) in both the right eye (OD) and the left eye (OS).

**Figure 2:**
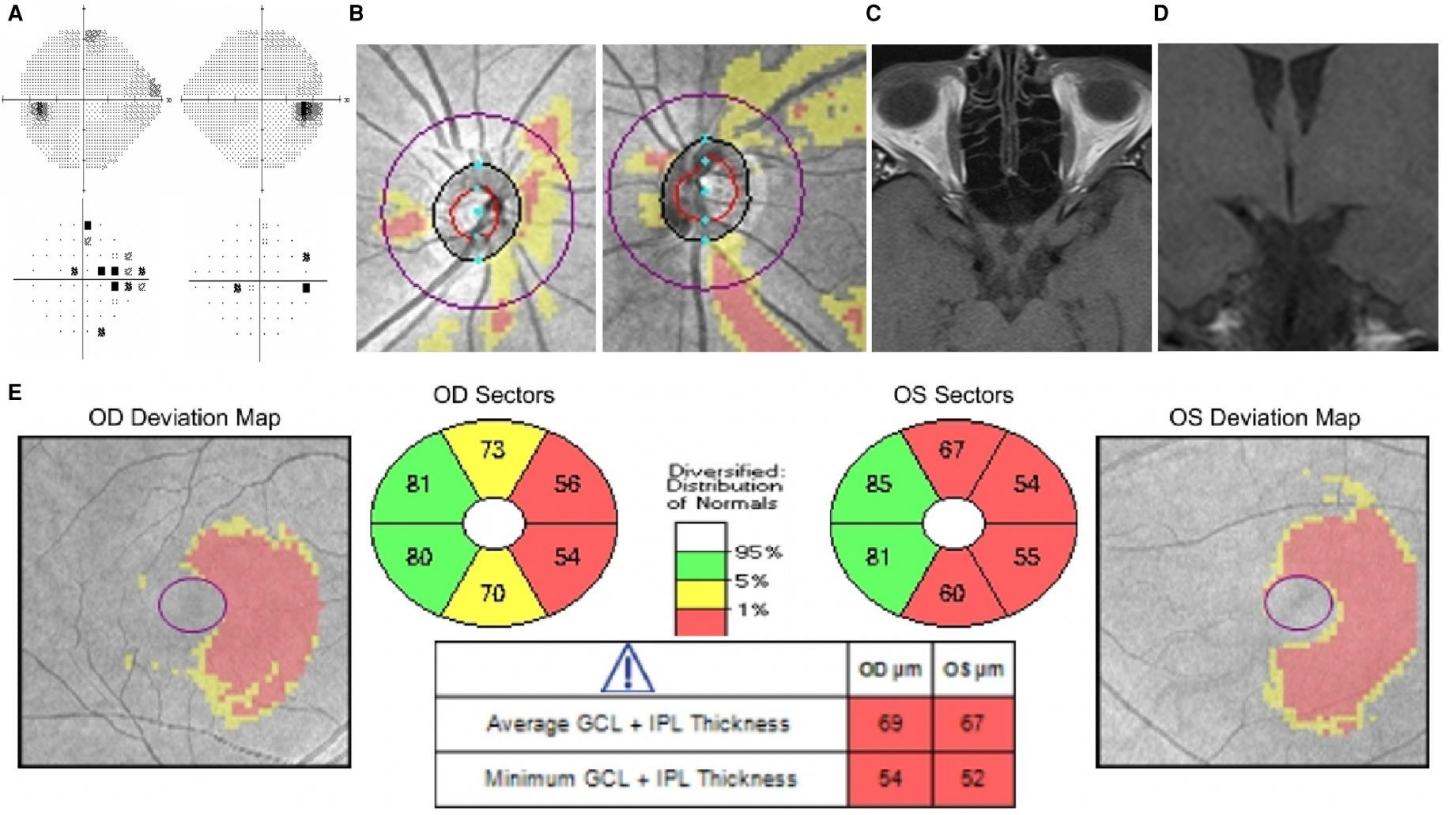
**Left OTS.** (**A**) Very subtle right HH seen on greyscale map and pattern deviation map, (**B**) band atrophy of the right optic disc and hourglass atrophy of the left optic disc, (**C**) thinning of the left optic tract seen on MRI axial T1 image and (**D**) coronal T1 image. (**E**) Left homonymous thinning of the GCL.

Figure 1 demonstrates an example of a typical patient with OTS, including incomplete incongruous HH, band and hourglass pRNFL thinning and homonymous thinning of GCL. Figure 2 demonstrates prominent pRNFL and homonymous GCL thinning, but a much more subtle pattern of homonymous VF loss.

### Neuroimaging findings

All patients underwent magnetic resonance imaging (MRI) of the brain/orbits and demonstrated abnormalities: 41% (*n* = 23) of patients had left OTLs, 55% (*n* = 31) had right OTLs and 4% (*n*  *=* 2) had bilateral involvement. Patients were divided into six categories based on their lesion aetiology: (i) space-occupying lesion (*n*  *=* 25; 45%), (ii) demyelinating disease (*n* = 11; 20%), (iii) ischaemia/haemorrhage (*n* = 9; 16%), (iv) non-specific optic tract atrophy (*n* = 8; 14%), (v) perinatal insult (*n* = 2; 4%) and (vi) trauma (*n* = 1; 2%). Patients with space-occupying lesions were further specified to have a meningioma (*n* = 7; 28%), glioma (*n* = 5; 20%), sellar/suprasellar lesion not-otherwise-specified (*n* = 4; 16%), cyst (*n*  *=* 2; 8%), cavernoma (*n* = 1; 4%), metastatic lesion (*n* = 1; 4%), lymphomatous lesion (*n* = 1; 4%), craniopharyngioma (*n* = 1; 4%), or unknown (*n* = 3; 12%). Causes of optic tract atrophy included idiopathic (*n* = 3; 38%), hereditary atrophy (*n* = 2; 25%), trauma (*n* = 2; 25%) and ischaemia (*n* = 1; 13%). Patients with optic tract atrophy caused by trauma or ischaemia differed from patients directly classified into the ischaemia or trauma categories, as the former primarily demonstrated atrophy on MRI while the latter presented with signs related to ischaemia/haemorrhage or trauma.

## Discussion

This study aimed to provide insight into neuro-ophthalmic examination findings, peripapillary OCT and ganglion cell analysis data and radiological characteristics of OTLs to help guide clinicians in ordering appropriate and timely imaging. We found that 78% of patients with HH had incongruous HH. We also identified that 48% demonstrated homonymous thinning on ganglion cell analysis, 41% had contralateral band and ipsilateral hourglass atrophy on pRNFL and 29% demonstrated contralateral RAPD. As such, this study demonstrates that OTS can present with heterogenous findings. Perimetry emerged as the most sensitive modality for detecting OTLs, followed by OCT and clinical examination. Etiologic subgroup analysis also revealed that patients with demyelinating lesions were most likely to present within the first 2 weeks of symptom onset (*P* = 0.0180). They were also the least likely to demonstrate band or hourglass atrophy on pRNFL (*P* = 0.0112), even when controlling for early presentation within 6 months (*P* = 0.0379).

Widely used in neuro-ophthalmology, formal perimetry is often the first investigation patients undergo when seeking care. Our findings have also revealed that it is a sensitive modality, with 98% of patients having VF defects. Among the patients with HH, a large proportion (78%) demonstrated incongruous HH. This is in contrast with a retrospective chart review by Kedar *et al.,*^5^ which suggests that only 50% of OTL patients with HH had incongruent HH. However, the findings of this study align with previous studies by Savino *et al.*^6^ and Rodriguez and Reddy,^7^ which propose that VF defects associated with OTLs are typically incongruous due to the relative separation of retinal fibres from each eye within the optic tract. Therefore, it is important for clinicians observing incomplete and incongruous VF defects in patients to consider the possibility of an OTL and carefully evaluate for other associated clinical findings.

Our study revealed that contralateral RAPD was only seen in 29% of patients. The subtlety of RAPD in some patients may have led to it being undetected, contributing to a low documentation rate. Furthermore, of the patients with contralateral RAPD, 80% demonstrated hemianopia while smaller VF defects like quadrantanopia were less common. Previous studies in optic neuropathies have shown that the severity of vision loss is proportional to the extent of ganglion cell loss, which in turn is reflected in the magnitude of the RAPD.^8,9^ This suggests that OTL patients with smaller VF defects may also have more subtle RAPD, highlighting the necessity of a thorough physical examination. Although no significant associations between lesion aetiology and presence of RAPD were found, only 14% of patients with optic tract atrophy had RAPD. We suspect that RAPD was less likely to be seen in some patients with non-specific atrophy of the optic tract because other parts of the visual pathway were also lesioned in these patients. For example, several of these patients were found to have extensive atrophy that involved the optic tract, chiasm, and/or optic nerves. Thus, it may also be reasonable to expect optic tract atrophy and/or more extensive pathology to be the culprit of OTS in patients without an RAPD.

RNFL and GCL findings are becoming increasingly relevant diagnostic modalities, with contralateral band and ipsilateral hourglass thinning on pRNFL and homonymous thinning on GCL being quite sensitive for diagnosing OTL.^10^ Previous studies have found that ganglion cell analysis is a more sensitive tool for detecting patients with OTL compared to global pRNFL values; however, pattern thinning of pRNFL with specific band/hourglass atrophy is a very specific finding for OTS.^11^ It is helpful to consider these parameters because despite the high prevalence of homonymous field defects, they are not specific to OTLs and can be seen in lesions affecting any part of the retrochiasmal pathway. Although it can be argued that similar patterns of superior and inferior pRNFL thinning have been reported in post-geniculate lesions, they often occur bilaterally in conjunction with nasal or temporal atrophy.^12^ Thus, when present, contralateral band and ipsilateral hourglass thinning remain highly specific indicators of OTS. This is particularly useful given that homonymous defects in OTL are usually incomplete, incongruous and can be quite subtle.^7^ Figure 2 demonstrates how inconspicuous the incongruous field loss can be and that examining ganglion cell analysis for presence of homonymous thinning and pRNFL for presence of band/hourglass atrophy can elucidate the aetiology of field loss. Of the 13 patients who did not demonstrate the expected congruous VF defects on perimetry, 69% had at least one OCT finding indicative of an OTL, highlighting the importance of RNFL and GCL in diagnosing OTS.

The use of both pRNFL and ganglion cell analysis is essential for diagnosing OTL, as only 16% of all patients had relevant findings on both modalities. Each modality identified an additional subset of cases, with 23% demonstrating only contralateral band and ipsilateral hourglass atrophy on pRNFL and 23% demonstrating only homonymous GCL thinning. Analysis also revealed that patients with certain aetiologies (i.e. demyelinating disease) were less likely to exhibit band or hourglass atrophy on pRNFL (*P*  *=* 0.0112). As patients with demyelinating disease were also most likely to present within 2 weeks (*P*  *=* 0.0180), the lack of pRNFL thinning may, in part, be related to earlier presentation of patients with demyelinating lesions. This is consistent with previous studies looking at other causes of anterior visual pathway insults, such as non-arteritic anterior ischaemic optic neuropathy,^13^ optic neuritis^14^ and glaucoma,^15^ which have shown that damage to GCL precedes that of pRNFL and while GCL thinning can be seen 4 weeks after the demyelinating attack, thinning on pRNFL can take up to 6 months to develop.^16,17^ However, this difference in pRNFL atrophy persisted even when limiting the analysis to patients who presented within 6 months of symptom onset, suggesting that the decreased likelihood of band or hourglass atrophy in demyelinating disease may reflect a true pathophysiological difference. This further suggests the necessity of using both pRNFL and ganglion cell analysis to diagnose OTS, especially in patients with a suspected demyelinating aetiology.

In addition to findings related to the classic OTS triad, this study highlighted several important clinical characteristics of patients with OTL. Visual acuity at presentation was slightly worse in the eye ipsilateral to the lesion (logMAR 0.20 ± 0.33) compared to the contralateral eye (logMAR 0.17 ± 0.29). This is consistent with findings by Savino *et al*., who reported greater acuity loss ipsilateral to the lesion in patients with OTLs. Although OTLs typically spare central visual acuity, the presence of vision loss may suggest the presence of additional lesions in other areas along the visual pathway.

Timing of presentation also varied by aetiology. Patients with OTS due to atrophy often presented later after symptom onset, on average 313.3 weeks. This may be due to the gradual decline of vision observed in cases of optic tract atrophy.^18^ In contrast, patients with demyelinating disease presented remarkably earlier, in keeping with the acute onset of symptoms in a demyelinating attack.^19^

Age at initial presentation also differed by lesion type. Patients with demyelinating disease, ischaemia/haemorrhage, perinatal insult and trauma as causes of OTS were on average younger than 50 years, whereas patients with space-occupying lesions and optic tract atrophy were older than 50 years. This is expected as demyelinating diseases such as multiple sclerosis usually present during the second, third and fourth decades of life^20^. Patients in early adulthood are more likely to engage in risk-seeking behaviours, which could account for higher prevalence of traumatic OTS in younger age group,^21^ and consequences of perinatal insult are more likely to be discovered early in life.

Different aetiologies of OTS also produced several distinct presenting symptoms: 56% of patients with ischaemia/haemorrhage had focal neurological symptoms, likely due to extensive damage in the vascular distribution of the affected vessel,^22^ while 24% of patients with space-occupying lesions presented with headache likely due to raised intracranial pressure and/or dural irritation.^23^

This study had several limitations. The incidence of OTS could not be determined as participants were recruited retrospectively. Furthermore, there was substantial variability in the aetiology of OTS and the number of patients in each category, making specific statistical analysis about each lesion type limited. Additional research powered by larger sample sizes is warranted.

Overall, OTLs can be difficult to diagnose and do not always present with classic triad. A high index of suspicion for OTL should be maintained in patients with unexplained mild RAPD, incongruous and partial homonymous VFs, presence of band and/or hourglass atrophy on peripapillary OCT and/or homonymous thinning on ganglion cell analysis. Clues on clinical history and testing may be suggestive of underlying lesion aetiology. Appropriate neuroimaging should be ordered in all patients with unknown aetiology of OTS and should be performed emergently in patients with concomitant new headaches or focal neurological deficits.

## Supplementary Material

fcaf354_Supplementary_Data

## Data Availability

Anonymized data not published within this article will be made available by request from any qualified investigator.
