# Supplementary material for: Diagnosis and etiologic classification of optic tract lesions
Source: Brain Commun. 2025 Sep 19;7(5):fcaf354. doi: 10.1093/braincomms/fcaf354 (PMC12477588; doi:10.1093/braincomms/fcaf354)
Supplement: fcaf354_Supplementary_Data [file fcaf354_supplementary_data.docx]

**Supplementary Table 1: Clinical Characteristics and Imaging Findings of Patients with Optic Tract Lesions at Presentation**

| **Patient**  **No.** | **Gender** | **VF Defect** | **OCT Pattern** | **Ganglion Cell Analysis Pattern** | **Laterality** | **MRI Findings/Final Diagnosis** |
| --- | --- | --- | --- | --- | --- | --- |
|  | | Space Occupying Lesions | | | | |
| 1 | F | Homonymous hemianopia (left) | Bowtie (OS) and hourglass (OD) | Junctional | Right | Suprasellar cistern lesion |
| 2 | F | Homonymous hemianopia (left) | Bowtie (OS) | Junctional | Right | Skull base meningioma |
| 3 | M | Junctional scotoma (left) | Bowtie (OS) and hourglass (OD) | Junctional | Right | CNS lymphoma lesion |
| 4 | F | Homonymous hemianopia (left) | Nonspecific | Nonspecific | Right | Sellar and cavernous sinus lesion |
| 5 | F | Homonymous quadrantanopia (right, inferior) | Hourglass (OS) | Homonymous | Left | Pituitary lesion |
| 6 | M | Homonymous hemianopia (right) | Bowtie (OD) and hourglass (OS) | Junctional | Left | Sellar lesion |
| 7 | M | Homonymous hemianopia (left) | Unreliable | No GCA performed | Right | Optic tract glioma |
| 8 | F | Homonymous hemianopia (right) | No pattern | Nonspecific | Left | Skull base meningioma |
| 9 | F | Bitemporal (left) | No pattern | Monocular diffuse | Right | Frontotemporal meningioma |
| 10 | F | Homonymous hemianopia (left) | Bowtie (OS) and hourglass (OD) | Nonspecific | Right | Anterior clinoid and cavernous sinus meningioma |
| 11 | M | Homonymous hemianopia (right) | Bowtie (OD) and hourglass (OS) | Nonspecific | Left | Craniopharyngioma |
| 12 | F | Homonymous hemianopia (right) | Bowtie (OD) and hourglass (OS) | Nonspecific | Left | Pilocytic astrocytoma |
| 13 | F | Homonymous quadrantanopia (right, superior) | Bowtie (OD) and hourglass (OS) | Homonymous | Left | Arachnoid cyst |
| 14 | F | Homonymous hemianopia (left) | Nonspecific | Homonymous | Right | Cavernous sinus meningioma |
| 15 | F | Homonymous hemianopia (right) | Bowtie (OD) and hourglass (OS) | Nonspecific | Left | Cystic lesion (likely pituitary macroadenoma or Rathke’s cleft cyst) |
| 16 | F | Homonymous hemianopia (right) | Bowtie (OD) | No GCA performed | Left | Intracranial glioma |
| 17 | F | Homonymous hemianopia (left) | Nonspecific | Nonspecific | Right | Meningioma |
| 18 | F | Homonymous hemianopia (right) | Nonspecific | Nonspecific | Left | Planum sphenoidale meningioma |
| 19 | F | Homonymous hemianopia (left) | Bowtie (OS) and hourglass (OD) | No GCA performed | Right | Cavernoma |
| 20 | F | Homonymous hemianopia (left) | Bowtie (OS) and hourglass (OD) | Nonspecific | Right | Cystic lesion (likely pilocytic astrocytoma) |
| 21 | F | Homonymous hemianopia (left) | Nonspecific | No GCA performed | Right | Lesion in temporal lobe |
| 22 | M | Homonymous hemianopia (left) | Nonspecific | Homonymous | Right | Lesion from metastatic melanoma |
| 23 | M | Homonymous hemianopia (bilateral) | Nonspecific | Homonymous | Right | Lesion in mesial temporal lobe |
| 24 | F | Homonymous hemianopia (left) | Nonspecific | Nonspecific | Right | Intracranial mass lesion |
| 25 | F | Homonymous hemianopia (right) | Bowtie (OD) and hourglass (OS) | Nonspecific | Left | Pilocytic astrocytoma |
|  | | Demyelinating Disease | | | | |
| 26 | F | Junctional scotoma (left) | Nonspecific | Normal | Left | Demyelinating lesion |
| 27 | F | Homonymous quadrantanopia (left, inferior) | Nonspecific | No GCA performed | Right | Demyelinating lesion |
| 28 | F | Homonymous hemianopia (bilateral) | Nonspecific | Homonymous | Both | Demyelinating lesion |
| 29 | F | Homonymous quadrantanopia (left, inferior) | Nonspecific | Normal | Right | Demyelinating lesion |
| 30 | M | Homonymous hemianopia (right) | Nonspecific | Normal | Left | Demyelinating lesion |
| 31 | F | Homonymous hemianopia (left) | Nonspecific | Homonymous | Right | Demyelinating lesion |
| 32 | M | Homonymous quadrantanopia (left, inferior) | Nonspecific | Homonymous | Right | Demyelinating lesion |
| 33 | F | Homonymous quadrantanopia (right, superior) | Hourglass (OS) | Homonymous | Left | Demyelinating lesion |
| 34 | F | No defect | Nonspecific | Homonymous | Left | Demyelinating lesion |
| 35 | M | Homonymous quadrantanopia (left, superior) | Nonspecific | Normal | Right | Demyelinating lesion |
| 36 | M | Homonymous hemianopia (left) | Nonspecific | Homonymous | Right | Demyelinating lesion |
|  | | Ischemic/Hemorrhage | | | | |
| 37 | F | Homonymous hemianopia (left) | Nonspecific | Nonspecific | Right | Infarct due to brain injury |
| 38 | M | Homonymous hemianopia (right) | Nonspecific | No GCA performed | Left | Intraparenchymal hemorrhage due to infective endocarditis |
| 39 | F | Homonymous hemianopia (left) | Bowtie (OS) and hourglass (OD) | Homonymous | Right | Intraparenchymal hemorrhage due to trauma |
| 40 | F | Homonymous hemianopia (right) | Nonspecific | Nonspecific | Left | Infarct in the left occipito-parietal lobe |
| 41 | F | Homonymous hemianopia (left) | Bowtie (OS) | Homonymous | Right | Subacute hemorrhage |
| 42 | F | Homonymous hemianopia (right) | Bowtie (OD) and hourglass (OS) | No GCA performed | Left | Infarct in left internal capsule due to surgery |
| 43 | F | Homonymous hemianopia (left) | Bowtie (OS) and hourglass (OD) | Homonymous | Right | Encephalomalacia due to previous infarct |
| 44 | F | Homonymous hemianopia (left) | Bowtie (OS) and hourglass (OD) | Homonymous | Right | Thalamic arteriovascular malformation causing hemorrhage |
| 45 | F | Homonymous hemianopia (left) | No pattern | Homonymous | Right | Middle cerebral artery stroke |
|  | | Optic Tract Atrophy | | | | |
| 46 | F | Homonymous hemianopia (left) | Bowtie (OS) and hourglass (OD) | No GCA performed | Right | Optic tract atrophy |
| 47 | M | Bitemporal hemianopia (superior) | Not applicable (glaucoma) | Nonspecific | Both | Optic tract atrophy secondary to hereditary chiasmopathy |
| 48 | F | Homonymous quadrantanopia (right, inferior) | Nonspecific | No GCA performed | Left | Optic tract atrophy due to hereditary chiasmopathy |
| 49 | M | Homonymous hemianopia (right) | Nonspecific | No GCA performed | Left | Optic tract atrophy |
| 50 | M | Homonymous quadrantanopia (right, superior) | Bowtie (OD) and hourglass (OS) | Homonymous | Left | Optic tract atrophy |
| 51 | M | Homonymous quadrantanopia (right, inferior) | Bowtie (OD) and hourglass (OS) | Homonymous | Left | Optic tract atrophy secondary to distant trauma |
| 52 | M | Homonymous hemianopia (right) | Bowtie (OD) and hourglass (OS) | Homonymous | Left | Optic tract atrophy secondary to remote infarct |
| 53 | M | Homonymous hemianopia (left) | Bowtie (OD) | Homonymous | Right | Optic tract atrophy secondary to distant trauma |
|  | | Perinatal Insult | | | | |
| 54 | F | Homonymous hemianopia (right) | Bowtie (OD) and hourglass (OS) | Homonymous | Left | Perinatal insult |
| 55 | M | Homonymous quadrantanopia (left, inferior) | Bowtie (OS) and hourglass (OD) | Nonspecific | Right | Perinatal insult |
|  | | Trauma | | | | |
| 56 | F | Homonymous hemianopia (left) | Bowtie (OS) and hourglass (OD) | Homonymous | Right | Trauma |

**Supplementary Material: Code Generated for “Diagnosis and Etiologic Classification of Optic Tract Lesions”**

*Code for Categorical Variables (Ex. Comparing Lesion Etiology to Proportion of Patients Presenting within 2 Weeks of Symptom Onset)*

clear all;

close all;

% Counts

count_1_within_2_weeks = 6;

count_2_within_2_weeks = 4;

count_3_within_2_weeks = 0;

count_4_within_2_weeks = 0;

% Total counts for each group

total_1 = 9;

total_2 = 18;

total_3 = 7;

total_4 = 4;

% Create pairwise comparisons and perform Fisher's exact test

groups = {'1', '2', '3', '4'};

num_groups = length(groups);

p_values = NaN(1, num_groups);

for i = 1:num_groups

other_groups = setdiff(1:num_groups, i);

combined_count_within_2_weeks = 0;

combined_total = 0;

% Loop through other groups to sum counts and totals

for j = 1:length(other_groups)

combined_count_within_2_weeks = combined_count_within_2_weeks + eval(sprintf('count_%d_within_2_weeks', other_groups(j)));

combined_total = combined_total + eval(sprintf('total_%d', other_groups(j)));

end

% Create contingency table for group i vs. combined other groups

count_i_within_2_weeks = eval(sprintf('count_%d_within_2_weeks', i));

total_i = eval(sprintf('total_%d', i));

contingency_table = [

count_i_within_2_weeks, total_i - count_i_within_2_weeks;

combined_count_within_2_weeks, combined_total - combined_count_within_2_weeks

];

% Perform Fisher's exact test

[~, p_value] = fishertest(contingency_table);

% Store the p-value

p_values(i) = p_value;

end

% Adjust p-values using Bonferroni correction

adjusted_p_values = p_values * num_groups; % Bonferroni correction

adjusted_p_values(adjusted_p_values > 1) = 1; % Ensure p-values do not exceed 1

% Display adjusted p-values

disp('Adjusted p-values:');

disp(adjusted_p_values);

*Code for Continuous Variables (Ex. Comparing Lesion Etiology to Age of Presentation)*

clear all;

close all;

% Load data

input_data = 'May_6_OTL_Reorganized_ Etiology.xlsx';

ds = spreadsheetDatastore(input_data);

T = readtable(input_data);

% Select columns for analysis

ageGroup1 = T{1:11, 2};

ageGroup2 = T{13:37, 2};

ageGroup3 = T{39:47, 2};

ageGroup4 = T{49:50, 2};

ageGroup5 = T{52, 2};

ageGroup6 = T{54:61, 2};

% Combine data

ageGroups = {ageGroup1, ageGroup2, ageGroup3, ageGroup4, ageGroup5, ageGroup6};

% Initialize results

pValues = zeros(1, 6);

% Perform Kruskal-Wallis test for each age group compared to the others

for i = 1:6

% Current age group

currentGroup = ageGroups{i};

% Remaining age groups combined

remainingGroups = vertcat(ageGroups{[1:i-1, i+1:end]});

% Combine data for Kruskal-Wallis

data = [currentGroup; remainingGroups];

groupLabels = [ones(size(currentGroup)); 2*ones(size(remainingGroups))];

% Perform Kruskal-Wallis test

p = kruskalwallis(data, groupLabels, 'off'); % 'off' to suppress the table output

% Store p-value

pValues(i) = p;

% Display the p-value for the current comparison

disp(['p-value for Age Group ', num2str(i), ' vs. Others: ', num2str(p)]);

end

% Apply Bonferroni correction

alpha = 0.05;

corrected_alpha = alpha / 6;

% Display all p-values with Bonferroni correction

disp('All p-values with Bonferroni correction:');

disp(['Significance level after Bonferroni correction: ', num2str(corrected_alpha)]);

disp(pValues);

% Display which comparisons are significant after Bonferroni correction

significantComparisons = pValues < corrected_alpha;

disp('Significant comparisons after Bonferroni correction:');

disp(significantComparisons);
